# Energy of the Isolated Metastable Iron-Nickel FCC Nanocluster with a Carbon Atom in the Tetragonal Interstice

**DOI:** 10.1186/s11671-017-1919-x

**Published:** 2017-02-21

**Authors:** Natalya V. Bondarenko, Anatoliy V. Nedolya

**Affiliations:** Applied Physics Department, Zaporizhzhya National University, 66 Zhukovsky St., 69600 Zaporizhzhya, Ukraine

**Keywords:** Nanocluster, Potential barrier, Tetrahedral interstice, Molecular mechanics method, Iron, 61.46.-w, 61.46.Bc, 61.46.Km

## Abstract

The energy of the isolated iron-nickel nanocluster was calculated by molecular mechanics method using Lennard-Jones potential. The cluster included a carbon atom that drifted from an inside octahedral interstice to a tetrahedral interstice in $<\overset{-}{1}11>$ direction and after that in <222> direction to the surface. In addition, one of 14 iron atoms was replaced by a nickel atom, the position of which was changing during simulation.

The energy of the nanocluster was estimated at the different interatomic distances. As a result of simulation, the optimal interatomic distances of Fe-Ni-C nanocluster was chosen for the simulation, in which height of the potential barrier was maximal and face-centered cubic (FCC) nanocluster was the most stable.

It is shown that there were three main positions of a nickel atom that significantly affected nanocluster’s energy.

The calculation results indicated that position of the carbon atom in the octahedral interstice was more energetically favorable than tetrahedral interstice in the case of FCC nanocluster. On the other side, the potential barrier was smaller in the direction $<\overset{-}{1}11>$ than in the direction <022>.

This indicates that there are two ways for carbon atom to drift to the surface of the nanocluster.

## Background

Supersaturation as well as local physical changes related to the presence of another kind of atoms [1, 2] may be (or not be) a precondition for the formation of a new cluster. There is a possibility of spontaneous emergence of clusters without a nucleus, their growth, and self-organization of cluster groups into a crystal of new phase [3].

Usually, the nanostructured materials and nanoparticles are created from traditional metal alloys under the influence of the extreme conditions: extrusion, multiple phase transitions, laser surface treatment, metal particles deposition from the vapor phase, etc. [4–6].

As a result, the metastable phases can be obtained because of the high cooling rate, high degrees of deformation, or both [7, 8].

In any case, the obtained nanostructures are quasi-stable and change their properties over time because the mass transfer processes become favorable energetically [9–11]. Cluster’s formation and destruction processes are very fleeting, especially, when it is less than critical size (about 1 nm), and its crystalline structure is not formed yet.

That is why calculation of nanoclusters’ energy can help us to understand the cause of their instability under the influence of impurity atoms. Also, it may be useful for the nanocluster properties regulation using the other type of atoms.

## Methods

For the study, we chose a face centered cubic (FCC) Fe-Ni-C nanocluster containing 15 atoms. We assumed that such a cluster forms randomly at initial time and contains one carbon atom and one nickel atom that substitutes iron atom. The system was considered to be quasi-stable and quasi-isolated that is why it was only statics that we took into account when estimating energy changes using molecular mechanic method (MM+ algorithm) [12–15]. We chose the FCC cluster because all the atoms in it are located on the surface or form the surface, which simplified interpretation of calculation results.

We performed an evaluation of energy empirically using the solution of the Newton system of equations:

1 $$ {m}_i\frac{d^2\overrightarrow{r_i}(t)}{d{ t}^2}=-\frac{\partial U\left(\overrightarrow{r_i},\dots, \overrightarrow{r_n}\right)}{\partial \overrightarrow{r_i}}+\overrightarrow{F_i^{\mathrm{ex}}}, $$

where Lennard-Jones pair-potential was:

2 $$ U\left({r}_{i j}\right)=4{\varepsilon}_{kl}{\displaystyle \sum_{i< j}\left[{\left(\frac{\sigma_{kl}}{r_{i j}}\right)}^{12}-{\left(\frac{\sigma_{kl}}{r_{i j}}\right)}^6\right]} $$

and where ${\varepsilon}_{kl}=\sqrt{\varepsilon_{kk}{\varepsilon}_{ll}}$—the bond energy, and ${\sigma}_{kl}=\frac{\sigma_{kk}+{\sigma}_{ll}}{2}$—the measure of the atomic size, were calculated using Lorenz-Berthelot mixing rule of atoms of *k*th and *l*th classes [16–18]; ${F}_i^{\mathrm{ex}}$—the force that determines intermolecular interactions; and *r*_*i*_ and *r*_*j*_—the coordinates of the interacting atoms ${r}_{i j}=\left|\overrightarrow{r_i}-\overrightarrow{r_j}\right|$. The choice of Lennard-Jones potential was associated with the fact that the size of nanocluster was less than a critical size (less than 1 nm), and the random forming of FCC of similar structure did not mean that it was crystalline in every sense of the word because it was smaller than three coordination spheres of atoms. The similar approach was described in [19, 20] for iron-nickel nanoparticles but using the Monte Carlo method. We determined the nanocluster energy at the initial time, once it had formed.

Due to the fact that the energy in such calculations is determined up to a constant, we calculated the energy difference between the position of atom of carbon inside of the nanocluster (octahedral interstice) and the current position during its drift to the surface:

3 $$ \varDelta u= u(L)- u(0), $$

where *L* is a length of the carbon atom path and *u* is the specific potential energy. Position of the carbon atom in the central octahedral interstitial site (COIS) of a cluster was chosen as null (0) of the path length (L), conforming to central symmetry of the nanocluster. We made an assumption that nanocluster’s surroundings were symmetrical and the number of atoms here had effect on the total energy of nanocluster, but it was not affecting its energy changes essentially (about 2% changes for 4 × 4 × 4 nm nanocluster size).

**Fig. 1:**
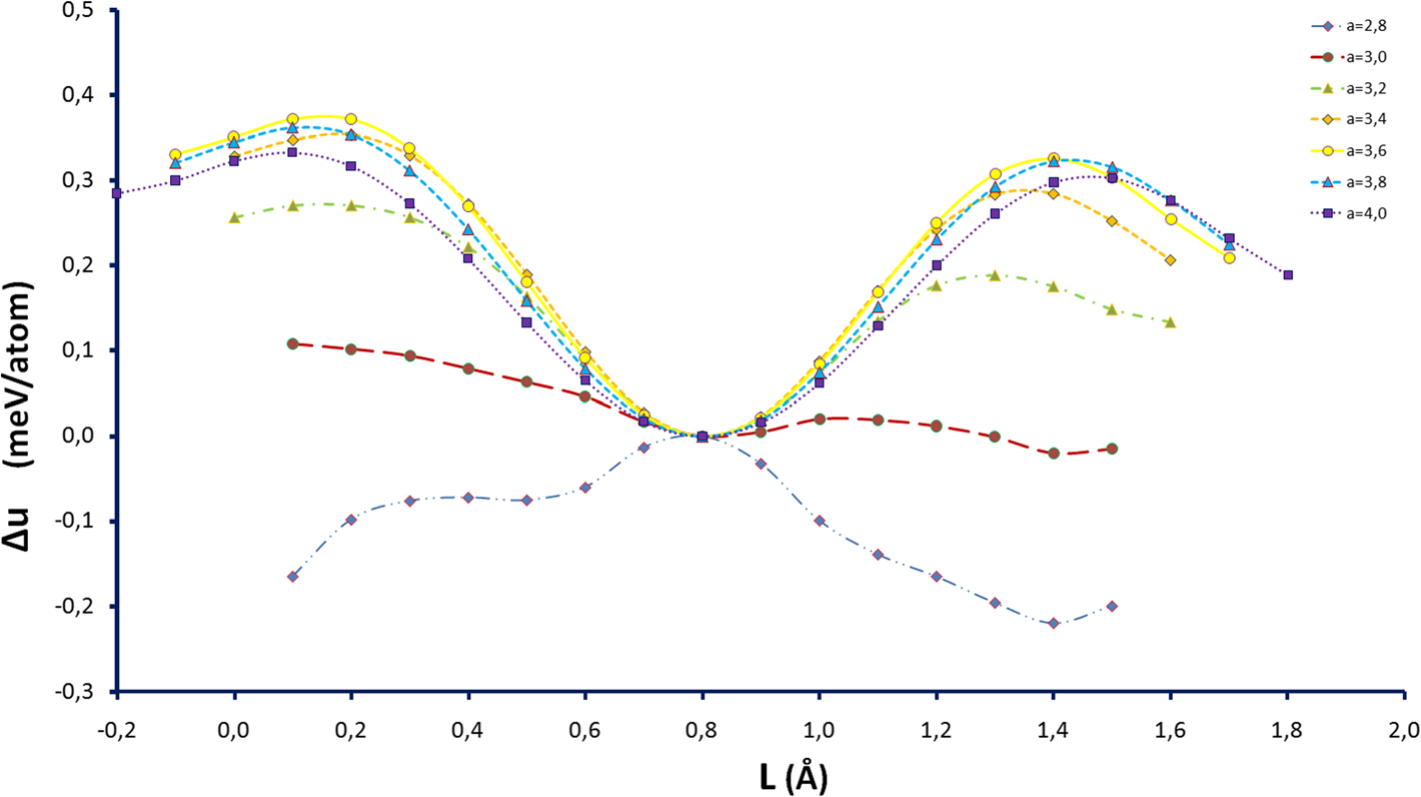
Change of specific energy iron-nickel FCC nanocluster depending on the distance between atoms (from 2.8 to 4.0 Å)

**Fig. 2:**
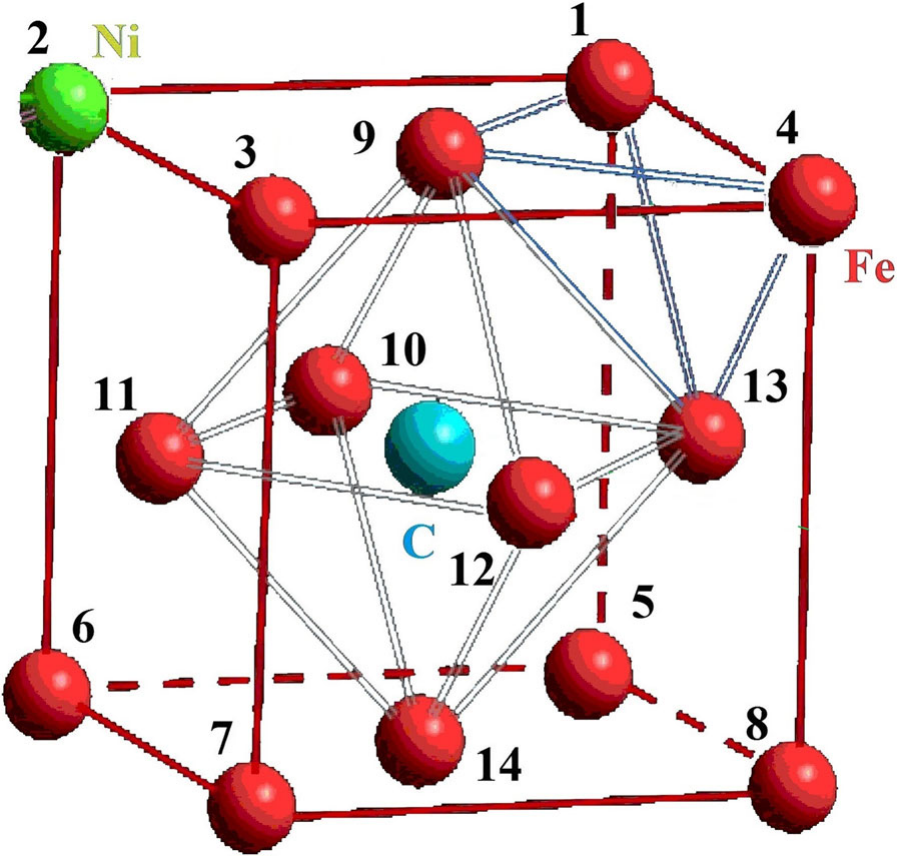
Numbering scheme of nickel atom positions in iron-nickel nanocluster

We considered the movement of carbon atom as a similar to the drift to surface due to the influence of surface energy nanocluster [21]. We examined every possible position of nickel atom, which replaced the iron atom, as an analog of random diffused jumps of nickel atom. Also, we selected the temperature of *T* = 300 K and the distances between atoms of 3.6 Å (angstroms) because the optimal interatomic distances of Fe-Ni-C nanocluster was chosen for simulation in which height of a potential barrier was maximal and FCC nanocluster was the most stable (see Fig. 1) [22–24]. We numbered their positions for convenience (see Fig. 2). In such a system, any changes of energy can be made only by changing positions of impurity atoms.

## Results and Discussion

**Fig. 3:**
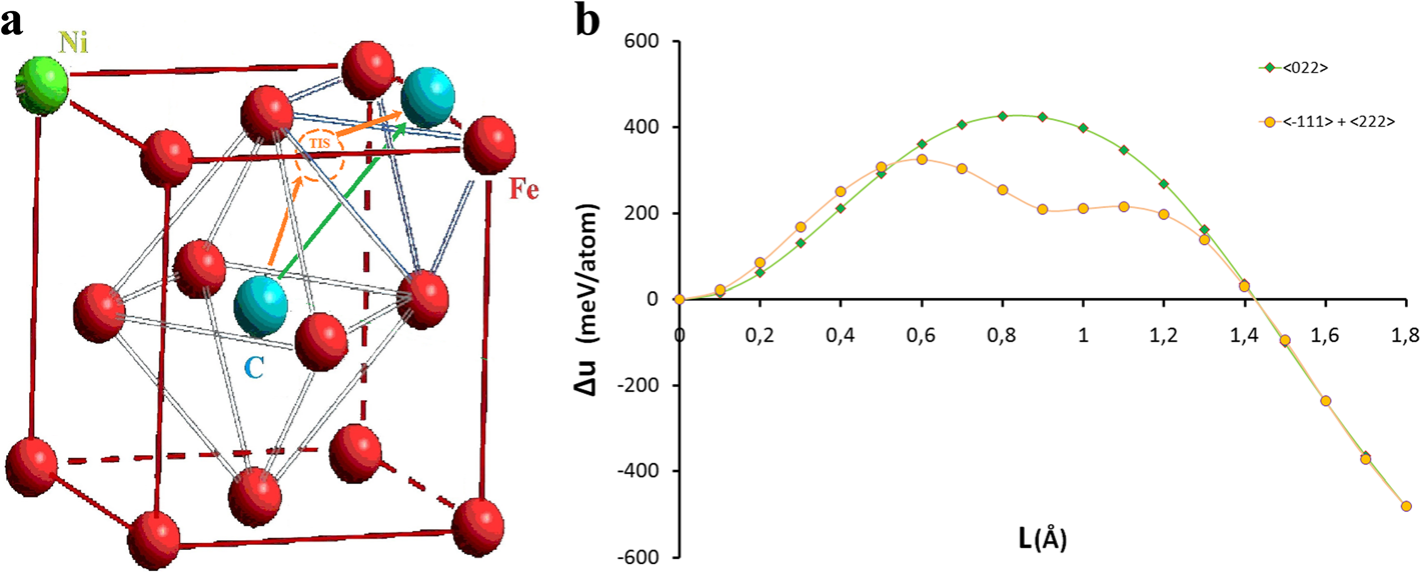
Scheme of carbon atom’s drift (**a**) and specific energy change of the iron-nickel nanocluster (**b**): *green arrow*, the direction <022>; *orange arrows*, $<\overset{-}{1}11>$ plus <222>

**Fig. 4:**
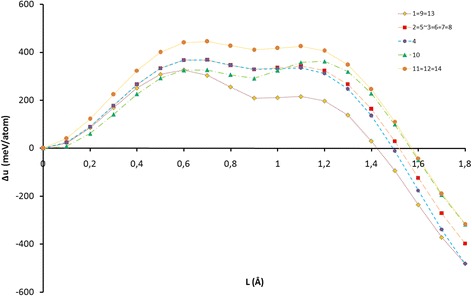
Change of specific energy of the iron-nickel nanocluster depending on the position of nickel atom

Nanocluster’s energy was calculated based on the location of the carbon atom, taking into account nickel atom’s position. We chose two directions of a carbon atom’s drift to the surface: direction <022> (green arrow) and the way $<\overset{-}{1}11>$ plus <222> (orange arrows) for calculation, which formed a triangle (Fig. 3a). Choice of the way $<\overset{-}{1}11>$ plus <222> was associated with the fact that it was able to pass through the tetrahedral interstice (TIS). Both directions were energetically favorable for a carbon atom because the cluster energy was almost twice smaller when the carbon atom was on the surface (*L = 1.8*) compared to its position in the central octahedral interstice (*L = 0*) due to influence of the surface as indicated in Fig. 4. However, in case when the carbon atom drifted towards <022> direction, the potential barrier (*Δ*) was higher than two potential barriers (*Δ*_1_, *Δ*_2_) in $<\overset{-}{1}11>$ plus <222> directions (see Fig 3b). We had calculated the energy of a FCC nanocluster of iron at all possible position of a nickel atom in order to determine its effect on the potential barriers’ height (see Table 1).

**Table 1:** Nanocluster energy at different positions of a carbon atom and a nickel atom

Equivalent positions of the Ni atom when a C atom occupies the TIS	Type of ratio PB highs	Δ*u*_1_ $\left(\frac{\mathsf{meV}}{\mathsf{atom}}\right)$	Δ*u*_2_ $\left(\frac{\mathrm{meV}}{\mathrm{atom}}\right)$	Δ*u*_min1,2_/Δ*u*_max1,2_ %	Δ $\left(\frac{\mathrm{meV}}{\mathrm{atom}}\right)$	Δ−Δ*u*_max1,2_ $\left(\frac{\mathrm{meV}}{\mathrm{atom}}\right)$	(Δ−Δ*u*_max1,2_)/Δ %
12 = = 11 = 14	a	440	425	3,5	505 535	65 95	13,0 18,0
4	b	370	335	9,5	430	60	14,0
2 = 3 = 5 = 6 = 7 = 8	b	370	345	7,0	460	90	19,5
1 = = 9 = 13	b	325	215	34,0	430 460	105 135	24,5 29,5
10	c	325	360	−10,0	505	145	29,0

Nanocluster energy at different positions of a carbon and a nickel atom at sequential drift of carbon atom in the direction <−111> and <222> to the surface (a) Δ*u*_1_ ≈ Δ*u*_2_ (with an accuracy of <5%), (b) Δ*u*_1_ > Δ*u*_2_, and (c) Δ*u*_1_ < Δ*u*_2_

*TIS* tetrahedral interstitial site, *PB* potential barrier

If the lowest potential barrier was in the direction of <022> when the nickel atom held positions 1 and 4, in the case of drift towards $<\overset{-}{1}11>$ plus <222>, potential barrier’s configuration was more complex.

There are three potential barriers’ ratios which a carbon atom can overcome using a tetrahedral interstice to reach the surface: (a) Δ_1_ ≈ Δ_2_; (b) Δ_1_ > Δ_2_, and (c) Δ_1_ < Δ_2_ (see Fig. 4).

**Fig. 5:**
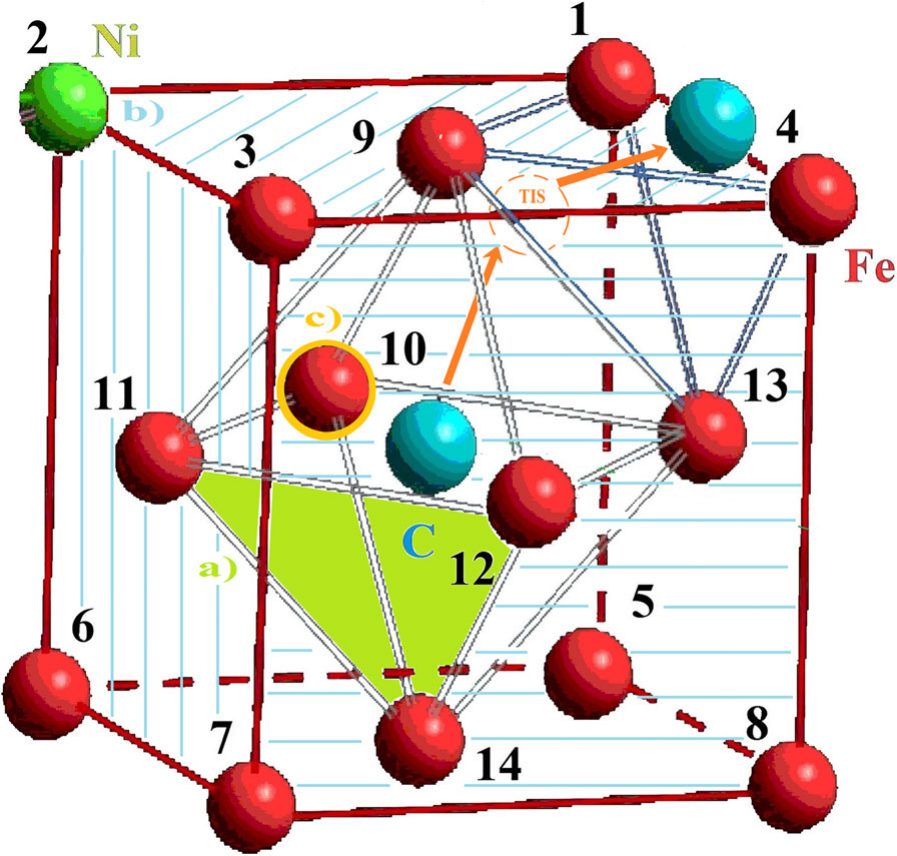
Scheme of nickel atom’s positions that affect the height of potential barriers of the tetrahedral interstice (a) Δ_1_ ≈ Δ_2_, *green*; (b) Δ_1_ > Δ_2_, *blue hatch*; and (c) Δ_1_ < Δ_2_, *orange*

Both of potential barriers with accuracy of 5% had an equal height when they corresponded to 11, 12, and 14 positions of nickel atom (see Fig. 5, green). In these cases, the heights of potential barriers on the way to the surface through tetrahedral interstice were 13–18% less than in the direction <022> (see Table 1a). Energy depth of tetrahedral interstices did not exceed 40 meV/atom or 11% between the maximum and the minimum. This position was the most stable of the three cases, although it was considerably unstable in comparison to the case when a carbon atom occupied the octahedral interstice.

In the second case (b), which included the majority of positions of a nickel atom (1 ÷ 9, 13), the first barrier was higher than the second potential barrier (see Fig. 5, blue hatch). This created an energy condition for carbon atom to drift to the surface in order to reduce the nanocluster energy. The energy advantage was from 14 to 20% in comparison to the direction <222>. For the carbon atom, the most energetically favorable was position 1 of a nickel atom.

There was a case where the height of the second potential barrier of tetrahedral interstice was larger than the first barrier’s by 10% (see Fig. 5, orange). In our opinion, although this height was significantly lower than the potential barrier of an octahedral interstice (by 29%), carbon atom’s drift to the surface through TIS was not energetically favorable because conditions for returning of a carbon atom to the central octahedral interstice were created.

## Conclusions

Thus, there are two ways for carbon atom to drift to the surface of the iron-nickel FCC nanocluster: short direction of <022> with high potential barrier and long direction $<\overset{-}{1}11>$ plus <222>, which potential barrier is lower by 13–29%. Carbon atom’s position in tetrahedral interstice is unstable, so it can be considered as a transit way of the carbon atom to the surface of the nanocluster.

The position of nickel atom affects the height of potential barriers and determines which of the two potential barriers of the tetrahedral interstice is higher. This can be considered as a method to control interstitial atom’s motion using the substitutional atom in nanocluster.

## Abbreviations

COIS: Central octahedral interstitial site
FCC: Face centered cubic
MM: Molecular mechanic
PB: Potential barrier
TIS: Tetrahedral interstitial site
